# Enhanced Singular Value Truncation Method for Non-Destructive Evaluation of Structural Damage Using Natural Frequencies

**DOI:** 10.3390/ma12071021

**Published:** 2019-03-28

**Authors:** Qiuwei Yang, Chaojun Wang, Na Li, Wei Wang, Yong Liu

**Affiliations:** 1 School of Civil Engineering, Shaoxing University, Shaoxing, Zhejiang 312000, China; yangqiuwei79@gmail.com (Q.Y.); qtwcj123@163.com (C.W.); lina@usx.edu.cn (N.L.); 2 State Key Lab Water Resources & Hydropower Engineering School, Wuhan University, Wuhan 430072, China; liuy203@whu.edu.cn

**Keywords:** non-destructive evaluation, structural damage, natural frequency, singular value truncation, multiple feedbacks, data noise

## Abstract

As natural frequencies can be easily and accurately measured, structural damage evaluation by frequency changes is very common in engineering practice. However, this type of method is often limited by data, such as when the available natural frequencies are very few or contaminated. Although much progress has been made in frequency-based methods, there is still much room for improvement in calculation accuracy and efficiency. To this end, an enhanced singular value truncation method is proposed in this paper to evaluate structural damage more effectively by using a few lower order natural frequencies. The main innovations of the enhanced singular value truncation method lie in two aspects: The first is the normalization of linear systems of equations; the second is the multiple computations based on feedback evaluation. The proposed method is very concise in theory and simple to implement. Two numerical examples and an experimental example are employed to verify the proposed method. In the numerical examples, it was found that the proposed method can successively obtain more accurate damage evaluation results compared with the traditional singular value truncation method. In the experimental example, it was shown that the proposed method possesses more precise and fewer calculations compared with the existing optimization algorithms.

## 1. Introduction

Structural damage often leads to changes in the dynamic response parameters of a structure. By testing the vibration parameters and observing their changes, structural damages can be monitored in a timely manner to avoid disastrous consequences. In recent decades, structural damage evaluation has become a key issue in the field of civil engineering, mechanical engineering, aerospace engineering and so on. The method based on natural frequency changes [1,2,3,4,5,6,7,8,9,10,11] is one of the mainstream methods for structural damage evaluation, since the natural frequencies are most easily and accurately measured in comparison with other dynamic characteristics of a structure. Messina et al. [3] proposed a damage detection method termed the multiple damage location assurance criterion by using the natural frequency sensitivity analysis. Yu et al. [4] made use of natural frequency perturbation theory and artificial neural network to detect small structural damage. Yang and Liu [5] proposed a frequency-based method with added masses to identify damages of the symmetrical structures. Khiem and Toan [6] proposed a method to calculate the natural frequencies of a multiple-cracked beam and detect an unknown number of multiple cracks from the measured natural frequencies. Ding et al. [7] presented an improved artificial bee colony algorithm for crack identification in beam structure. Krishnanunni et al. [8] defined an objective function using the frequency sensitivity equation and minimized it using a cuckoo search algorithm to evaluate structural damage. Choi and Han [9] studied frequency-based damage detection in cantilever beam by using a vision-based monitoring system with a motion magnification technique. Pan et al. [10] proposed a novel concept of noise response rate (NRR) to evaluate the sensitivity of each mode of the frequency shift to noise. It was shown that selecting vibration modes with low NRR values improves the prediction accuracy of frequency-based damage detection. Ercolani et al. [11] studied the inverse method of damage detection from the measurement of the first three natural frequencies of vibration on two experimental beams.

Although much progress has been made in frequency-based methods, there is still much room for improvement in the calculation accuracy and efficiency since the available natural frequencies are very few and contaminated. For the damage identification problem, the damaged elements in the structure are often only a small minority because the actual damage usually occurs only in a few local areas. This particularity of damage identification has not been fully utilized in the previous frequency-based methods. In this paper, an enhanced singular value truncation (ESVT) method is proposed for structural damage evaluation by using only a few natural frequencies. Central to the proposed method is the normalization of linear systems of equations and the multiple computations based on feedback evaluation. The above particularity of damage detection is fully utilized in the proposed procedure by removing many undamaged elements in each computation according to the feedback evaluation. This operation can significantly reduce the computational complexity and obtain more accurate damage evaluation results. The presentation of this work is organized as follows. In Section 2, the natural frequency sensitivity theory is brief reviewed and then an enhanced singular value truncation method is proposed for structural damage evaluation. Two numerical examples and an experimental example are used to demonstrate the feasibility and superiority of the developed method in Section 3 and Section 4, respectively. From the numerical results, it was found that the proposed method can successively obtain more accurate damage evaluation results compared with the traditional singular value truncation method. From the experimental results, it was shown that the proposed method possesses more precise and fewer calculations compared with the existing optimization algorithms. The conclusions of this work are summarized in Section 5.

## 2. Theoretical Development

### 2.1. Natural Frequency Sensitivity for Damage Detection

As is well known, the low-order natural frequencies of structural vibration can be easily and accurately measured in engineering practice. Thus the natural frequency is the most commonly used parameter in structural model updating or damage detection. In this section, the basis for the natural frequency sensitivity technique [1,2,3,4,5] is briefly reviewed. According to the vibration theory, the modes of structural free vibration can be obtained theoretically by solving the following generalized eigenvalue problem:

(1) $$K\phi_{j}=\lambda_{j}M\phi_{j}$$

where M and K are the mass and stiffness matrices of the structure, and $\lambda_{j}$ and $\phi_{j}$ are the jth eigenvalue and eigenvector, respectively. Note that the eigenvalue $\lambda_{j}$ can be obtained from the corresponding natural frequency $f_{j}$ by

(2) $$\lambda_{j}={\left(2\pi \cdot f_{j}\right)}^{2}$$

Generally, the mass matrix Mis assumed constant in model updating or damage detection. Then the first-order sensitivity of the jth eigenvalue $\lambda_{j}$ can be computed by

(3) $$\frac{\partial \lambda_{j}}{\partial x_{i}}=\phi_{j}^{T}K_{i}\phi_{j}$$

where $x_{i}$ and $K_{i}$ are the ith elemental stiffness perturbed parameter (also called as damage parameter) and stiffness matrix, respectively. The goal of model updating or damage detection is to obtain the values of these stiffness perturbed parameters by the changes between the measured eigenvalues and the theoretical eigenvalues. Assuming $\lambda_{j}^{*}$ is the jth measured eigenvalue, the eigenvalue change $\Delta \lambda_{j}$ can be calculated as

(4) $$\Delta \lambda_{j}=\lambda_{j}^{*}-\lambda_{j}$$

On the other hand, the eigenvalue change $\Delta \lambda_{j}$ can be approximated using Taylor’s series expansion and linear superposition principle as

(5) $$\Delta \lambda_{j}=\sum_{i=1}^{N}x_{i}\frac{\partial \lambda_{j}}{\partial x_{i}}$$

where N is the number of total elements in structural finite element model (FEM). For m measured eigenvalues, the first-order sensitivity equation of natural frequencies can be obtained as

(6) A⋅x=b

(7) $$A=\left[\begin{array}{ccc} \frac{\partial \lambda_{1}}{\partial x_{1}} & \cdots & \frac{\partial \lambda_{1}}{\partial x_{N}}\\ \vdots & \ddots & \vdots \\ \frac{\partial \lambda_{m}}{\partial x_{1}} & \cdots & \frac{\partial \lambda_{m}}{\partial x_{N}} \end{array}\right]$$

(8) $$x=\left\{\begin{array}{c} x_{1}\\ \vdots \\ x_{N} \end{array}\right\}$$

(9) $$b=\left\{\begin{array}{c} \Delta \lambda_{1}\\ \vdots \\ \Delta \lambda_{m} \end{array}\right\}$$

By solving the linear Equation (6), the unknown stiffness perturbed parameters $\alpha_{i}$ can be obtained, which will be used for model updating or damage evaluation. For example, the generalized inverse [12,13,14,15] is used in many cases to compute x in Equation (6), that is

(10) $$x=A^{+}b$$

where the superscript “+” denotes the Moore–Penrose generalized inverse [16]. 

### 2.2. Enhanced Singular Value Truncation Method

In engineering practice, only a few lower order natural frequencies with noise can be obtained through structural vibration testing [17,18,19,20]. Thus the results obtained by Equation (10) are often very unstable and inaccurate. This leads to the failure of model updating and damage detection. Therefore it is very necessary to develop a new computational method to compute the stiffness perturbed parameters more reliably. Traditionally, the singular value truncation (SVT) method [21,22,23,24,25,26,27,28] can be used to replace the generalized inverse to solve Equation (6) more effectively. However, as will be shown in the next example, the results obtained by the common SVT are still undesirable for many cases. In view of this, an ESVT method is proposed in this section to obtain more accurate x for structural damage evaluation. The proposed ESVT method is very concise in theory and very easy in calculation. The innovations of the ESVT method lie in two aspects: (1) Normalization of linear systems of equations; (2) multiple computations based on feedback evaluation. The ESVT method is illustrated in detail as follows.

Using the similar idea of the total least squares method [29,30,31,32,33], the linear systems of Equation (6) can be normalized by the division operation as

(11) $$A^{*}x=1_{v}$$

(12) $$1_{v}=\left\{\begin{array}{c} 1\\ \vdots \\ 1 \end{array}\right\}$$

(13) $$A^{*}=\left[\begin{array}{ccc} \frac{a_{11}}{b_{1}} & \cdots & \frac{a_{1N}}{b_{1}}\\ \vdots & \ddots & \vdots \\ \frac{a_{m1}}{b_{m}} & \cdots & \frac{a_{mN}}{b_{m}} \end{array}\right]$$

where $a_{ij}$ denotes the (i,j)th coefficient of A and $b_{i}$ denotes the ith coefficient of b in Equation (6). The advantage of this normalization is that all errors, including measurement errors and model errors, are placed in the new coefficient matrix $A^{*}$. It will be found that this normalization process can improve the accuracy and robustness of the solution for the linear systems of equations. After the normalization process, Equation (11) can then be solved through the singular value truncation technique as follows. Performing the singular value decomposition on $A^{*}$ in Equation (11), one has

(14) $$U\Lambda V^{T}\cdot x=1_{v}$$

(15) $$U=[u_{1},u_{2},\cdots ,u_{n}]$$

(16) $$V=[v_{1},v_{2},\cdots ,v_{N}]$$

(17) $$\Lambda =\left[\begin{array}{cc} Z & 0\\ 0 & 0 \end{array}\right],Z=diag(\sigma_{1},\sigma_{2},\cdots ,\sigma_{t})$$

where U and V are the orthogonal matrices, and $\sigma_{1},\sigma_{2},\cdots ,\sigma_{t}$ are the nonzero singular values of $A^{*}$ with $\sigma_{1}\geq \sigma_{2}\geq \cdots \geq \sigma_{t}$. By ignoring some smaller singular values, the singular value truncation solution of x for the first time can be obtained from Equation (14) as

(18) $$x=(\sum_{y=1}^{s}\sigma_{y}^{-1}v_{y}u_{y}^{T})\cdot 1_{v}$$

where s is the number of remained singular values, s≤t. The suitable value of s is determined by the L-curve method [34,35,36]. The main steps of the L-curve method are as follows: (1) Compute all possible solutions of x by Equation (18) when s is taken from 1 to t. (2) For each solution of x, calculate the 2-norm of x and Ax−b(or $A^{*}x-1_{v}$). (3) Draw the scatter plot with ${\left\|Ax-b\right\|}_{2}$ as abscissa and ${\left\|x\right\|}_{2}$ as ordinate (${\left\|\cdot \right\|}_{2}$ denotes the 2-norm). (4) Connect the resulting scatters with straight lines to form the L-curve. (5) Determine the suitable value of s according to the inflection point of the L-curve. The L-curve method will be further illustrated in Section 3.1.

For the damage evaluation problem, the perturbed elements in the FEM due to damages are often only a small minority. This particularity of damage detection problem has not been fully utilized in the published frequency-based algorithms. This particularity results in the existence of a large number of coefficients close to zero in the x obtained by Equation (18). Thus these coefficients close to zero in x should be seen as a product of data noise and set to zeros to simplify the equation (11) for the next recalculation. Generally, those values in x that satisfy $\frac{x_{i}}{\max(x)}\leq 0.05$ should be deemed to correspond to those undamaged elements in the structure. Then Equation (11) can be further simplified for the recalculation by removing some column vectors in $A^{*}$ and coefficients in x corresponding to those undamaged elements. That is

(19) $$A_{2}^{*}\cdot x^{\prime }=1_{v}$$

where $A_{2}^{*}$ is the remained matrix of $A^{*}$ after removing some column vectors related to those undamaged elements, $x_{2}$ is the remained vector of x after removing the corresponding coefficients. From Equation (19), the solution of $x^{\prime }$ can be obtained again using the similar singular value truncation progress between Equations (14) and (18) as

(20) $$x^{\prime }=(\sum_{y=1}^{s^{\prime }}\sigma_{y}^{{}^{\prime }-1}v_{y}^{\prime }u_{y}^{{}^{\prime }T})\cdot 1_{v}$$

Note that the result obtained by Equation (20) is maybe still not the final solution. When $x^{\prime }$ is the same as the corresponding coefficients in x, the $x^{\prime }$ in Equation (20) is the final solution of the damage parameters. If not, the above recalculation process should be repeated and the new solution $x^{\prime \prime }$ of the stiffness perturbed parameters can be obtained. The above process should be repeated until the solutions of the two adjacent cases are exactly the same (for example, $x^{\prime \prime }=x^{\prime }$). At the last, structural damage evaluation can be carried out according to the final result. In the above process, it is important to note that the computational complexity of each computation in ESVT gradually decreases since the number of unknowns decreases gradually. 

## 3. Numerical Examples

### 3.1. A Truss Structure

**Figure 1:**
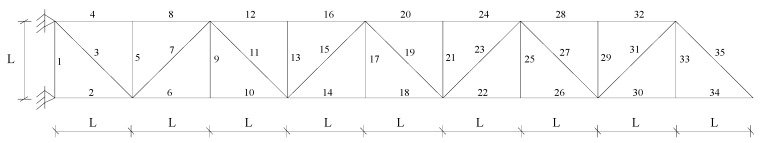
A cantilever truss structure (L = 0.5 m).

A cantilever truss structure as shown in Figure 1 was taken as the numerical example to demonstrate the effectiveness of the proposed method. The basic parameters of the structure were as follows: Young’s modulus E=200 GPa, density $\rho =7.8\times 10^{3}\;\mathrm{kg}/m^{3}$, and cross-sectional area $A=3.14\times 10^{-4}\;m^{2}$. Two damage cases were studied in the example. The first one was a single damage case where element 10 has a 20% stiffness reduction. The second was a multiple damage case where elements 7 and 18 have 15% and 20% stiffness reductions, respectively. Only the first six natural frequencies (shown in Table 1) of the undamaged and damaged structures were used in the structural damage evaluation. 

**Figure 2:**
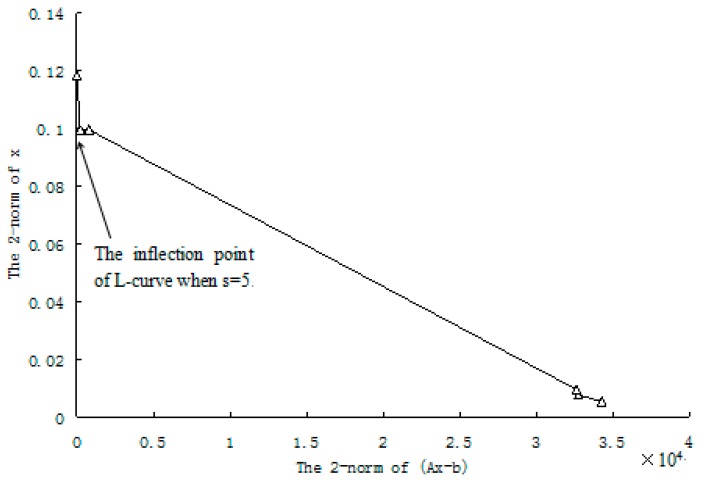
The L-curve used to determine the number of remained singular values in singular value truncation (SVT) for case 1.

**Figure 3:**
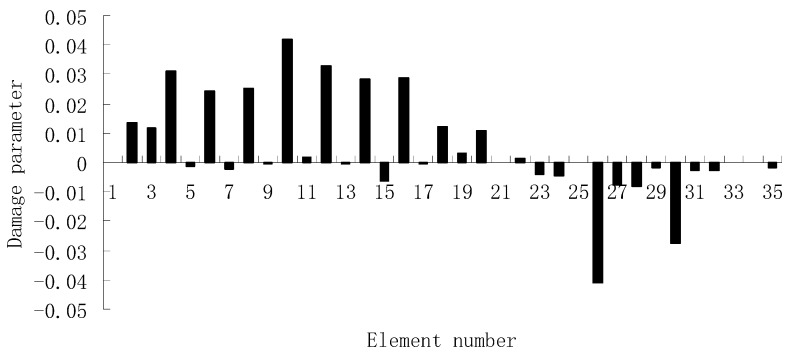
Damage evaluation result by the traditional SVT method for case 1 (element 10 had 20% stiffness reduction).

**Figure 4:**
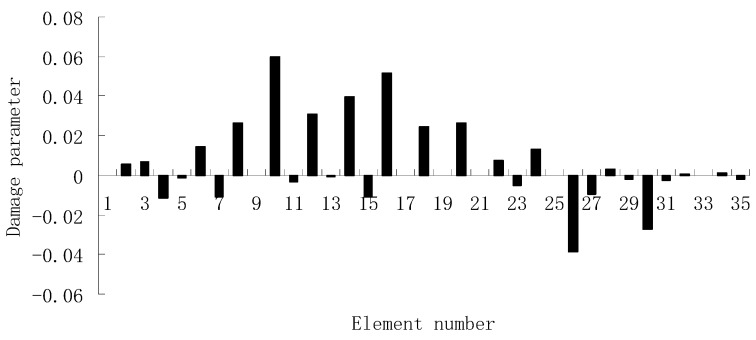
Damage evaluation result by the first computation of enhanced singular value truncation (ESVT) for case 1 (element 10 had 20% stiffness reduction).

**Figure 5:**
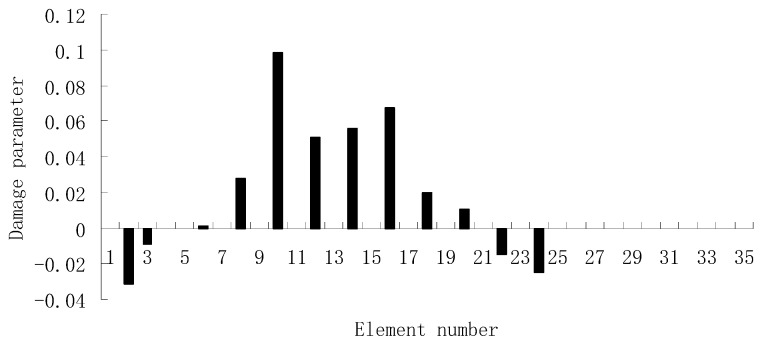
Damage evaluation result by the second computation of ESVT for case 1 (element 10 had 20% stiffness reduction).

**Figure 6:**
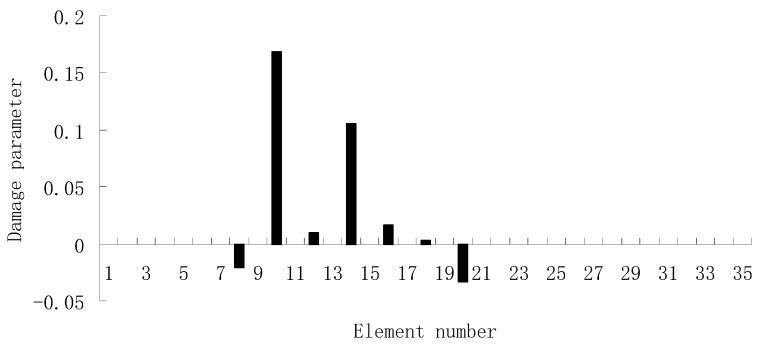
Damage evaluation result by the third computation of ESVT for case 1 (element 10 had 20% stiffness reduction).

**Figure 7:**
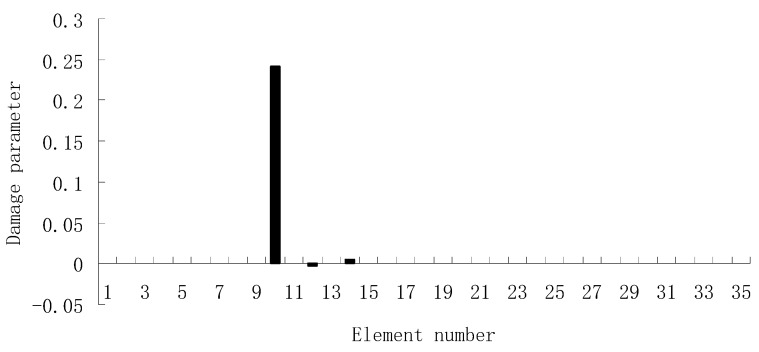
Damage evaluation result by the fourth computation of ESVT for case 1 (element 10 had 20% stiffness reduction).

**Figure 8:**
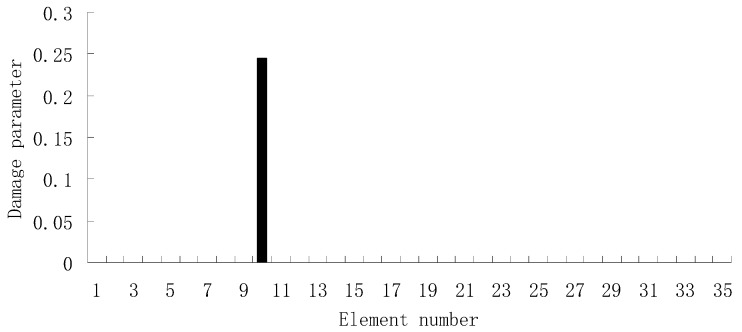
Damage evaluation result by the fifth computation of ESVT for case 1 (element 10 had 20% stiffness reduction).

For each of damage cases, the evaluation results obtained by the SVT and ESVT are both given to illustrate the superiority of the ESVT method. For case 1, the SVT method was firstly employed to compute the damage parameters. As stated before, the suitable value of s in the computation process is determined by the L-curve as shown in Figure 2. Note that the scatters from right to left in Figure 2 correspond to s = 1, s = 2, etc. One can see from Figure 2 that the inflection point of the L-curve just corresponded to s = 5. Subsequently Figure 3 presents the damage evaluation result obtained by SVT method with s = 5. One can see from Figure 3 that the result was not satisfactory since element 10 cannot be uniquely determined as the damage element. Using the proposed ESVT method, Figure 4, Figure 5, Figure 6, Figure 7 and Figure 8 give the damage evaluation results of the first to fifth calculations in ESVT. Apparently, the accuracy of damage evaluation result in Figure 4, Figure 5, Figure 6, Figure 7 and Figure 8 was improving gradually and Figure 8 was the final result. It can be seen from Figure 8 that, after five operations, element 10 could be uniquely determined as the damage element. It was thus shown that the proposed ESVT method can achieve higher evaluation accuracy than the traditional SVT method.

**Figure 9:**
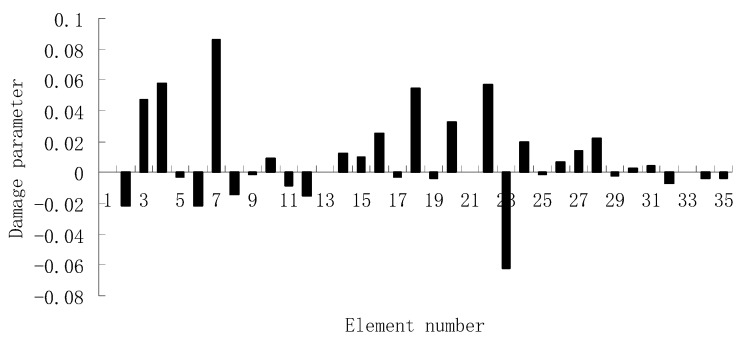
Damage evaluation result by the traditional SVT method for case 2 (elements 7 and 18 had 15% and 20% stiffness reductions).

**Figure 10:**
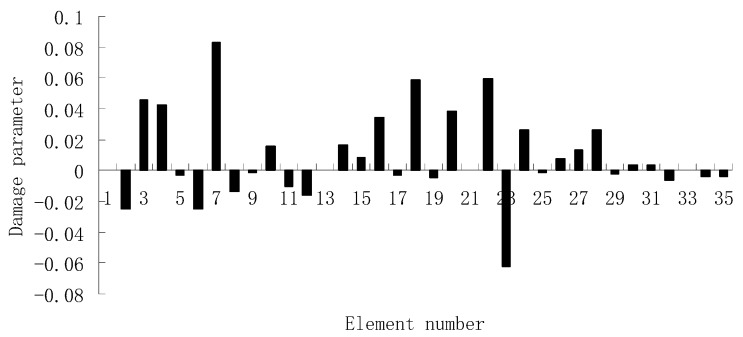
Damage evaluation result by the first computation of ESVT for case 2 (elements 7 and 18 had 15% and 20% stiffness reductions).

**Figure 11:**
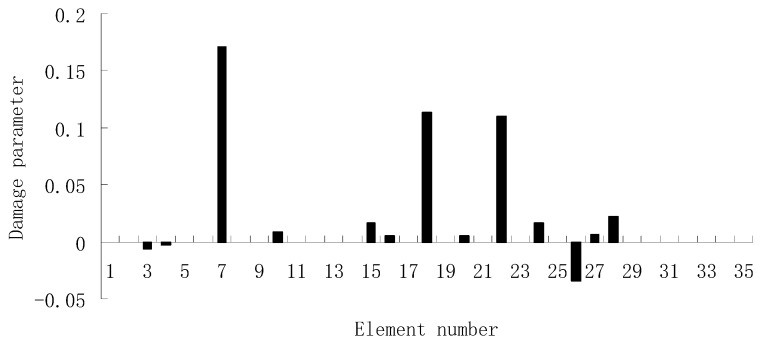
Damage evaluation result by the second computation of ESVT for case 2 (elements 7 and 18 had 15% and 20% stiffness reductions).

**Figure 12:**
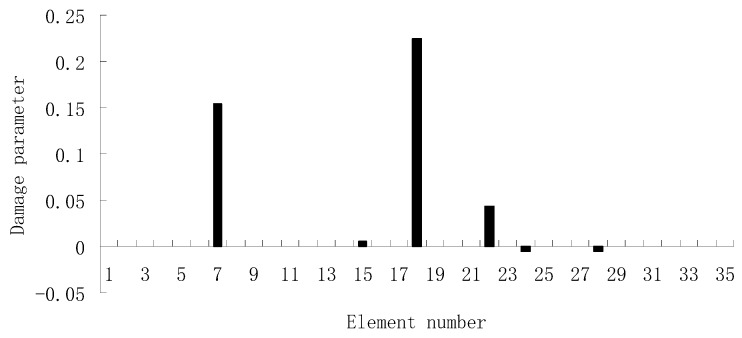
Damage evaluation result by the third computation of ESVT for case 2 (elements 7 and 18 had 15% and 20% stiffness reductions).

**Figure 13:**
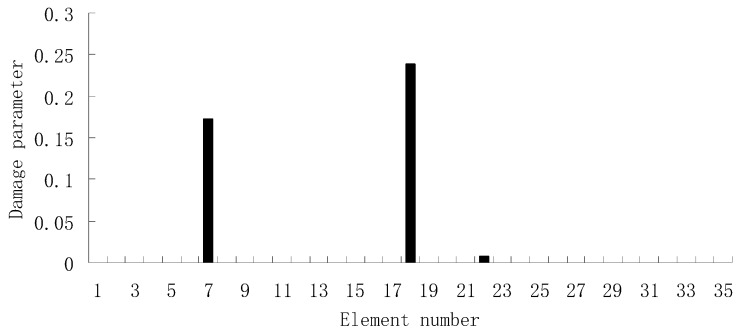
Damage evaluation result by the fourth computation of ESVT for case 2 (elements 7 and 18 had 15% and 20% stiffness reductions).

**Figure 14:**
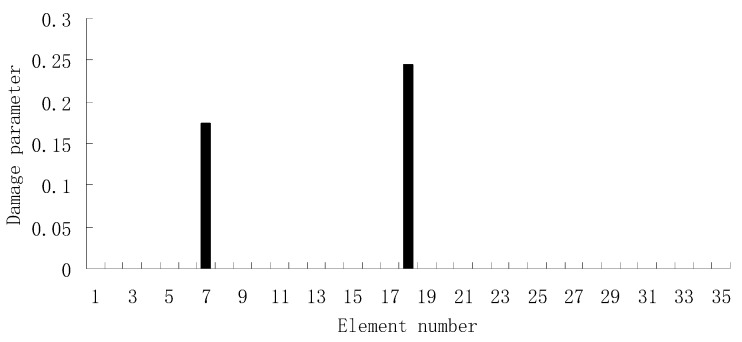
Damage evaluation result by the fifth computation of ESVT for case 2 (elements 7 and 18 had 15% and 20% stiffness reductions).

For the second damage case, Figure 9 presents the damage evaluation result obtained by the traditional SVT method. From Figure 9, it was found that the result was not satisfactory since many elements besides 7 and 18 were determined as the damaged elements. Using the proposed ESVT method, Figure 10, Figure 11, Figure 12, Figure 13 and Figure 14 provide the damage evaluation results of the first to fifth calculations in ESVT. It was clear that the accuracy of damage evaluation result in Figure 10, Figure 11, Figure 12, Figure 13 and Figure 14 was improving gradually and Figure 14 was the final result for this case. The final result of Figure 14 clearly indicated that elements 7 and 18 were the true damaged elements. These results again show that the proposed ESVT method can achieve higher evaluation accuracy than the traditional SVT method.

**Figure 15:**
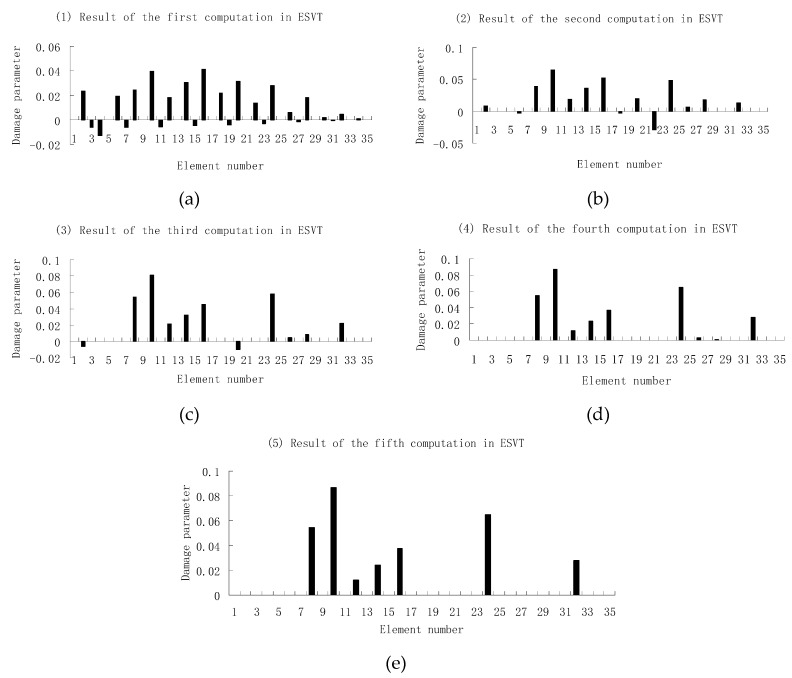
Damage evaluation results of the first to fifth computations in ESVT using the first three frequencies (element 10 had 20% stiffness reduction). (**a**) Result of the first computation in ESVT; (**b**) Result of the second computation in ESVT; (**c**) Result of the third computation in ESVT; (**d**) Result of the fourth computation in ESVT; (**e**) Result of the fifth computation in ESVT.

**Figure 16:**
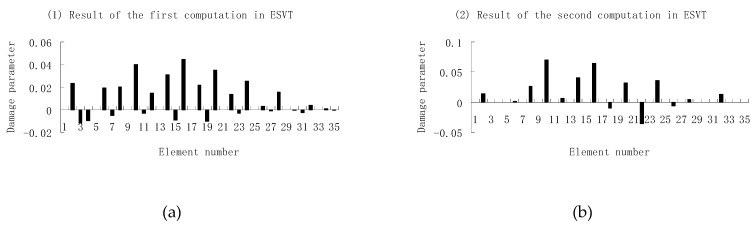

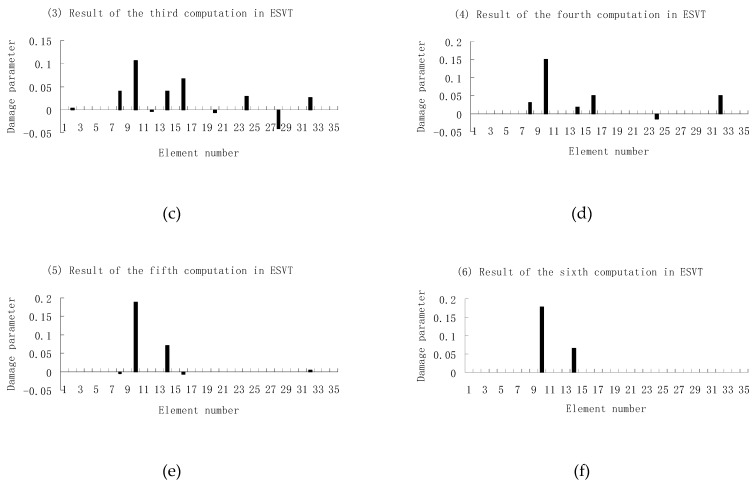
Damage evaluation results of the first to sixth computations in ESVT using the first four frequencies (element 10 had 20% stiffness reduction). (**a**) Result of the first computation in ESVT; (**b**) Result of the second computation in ESVT; (**c**) Result of the third computation in ESVT; (**d**) Result of the fourth computation in ESVT; (**e**) Result of the fifth computation in ESVT; (**f**) Result of the sixth computation in ESVT.

**Figure 17:**
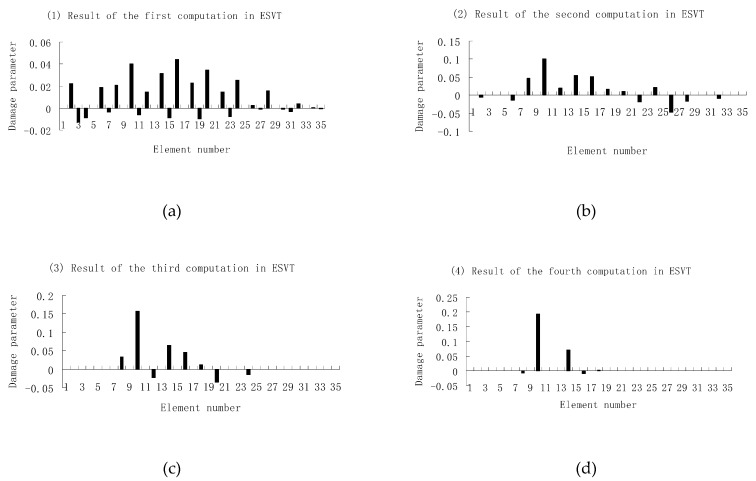

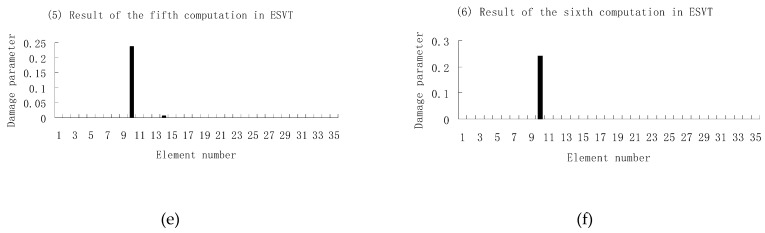
Damage evaluation results of the first to sixth computations in ESVT using the first five frequencies (element 10 had 20% stiffness reduction). (**a**) Result of the first computation in ESVT; (**b**) Result of the second computation in ESVT; (**c**) Result of the third computation in ESVT; (**d**) Result of the fourth computation in ESVT; (**e**) Result of the fifth computation in ESVT; (**f**) Result of the sixth computation in ESVT.

Next, Figure 15, Figure 16 and Figure 17 present damage evaluation results using the first three, four and five frequencies to investigate the effect of the frequency number on the calculation results. From Figure 15, one can see that the result was not satisfactory when only three frequencies were used since element 10 cannot be uniquely determined as the damaged element in the final result of Figure 15e. From Figure 16, the result was also not satisfactory when only four frequencies were used since element 10 cannot be uniquely determined as the damage element in the final result of Figure 16f. When five frequencies were used, it can be seen from Figure 17 that the result was satisfactory since element 10 can be uniquely determined as the damaged element after six computations. It was thus shown that the results of damage evaluation become more accurate as the number of used frequencies increases. For this example, at least five frequencies were needed to obtain sufficient accurate damage evaluation results.

### 3.2. A Plate Structure

**Figure 18:**
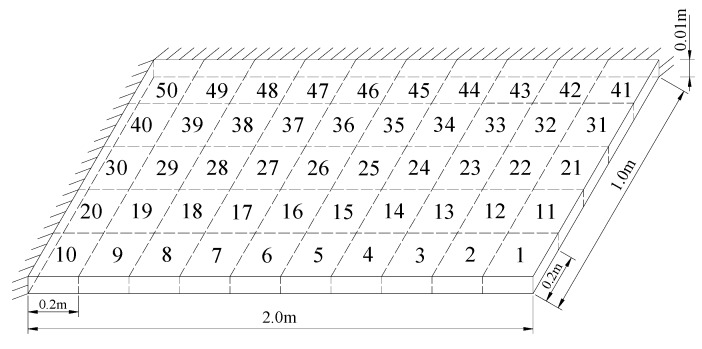
A plate structure.

**Figure 19:**
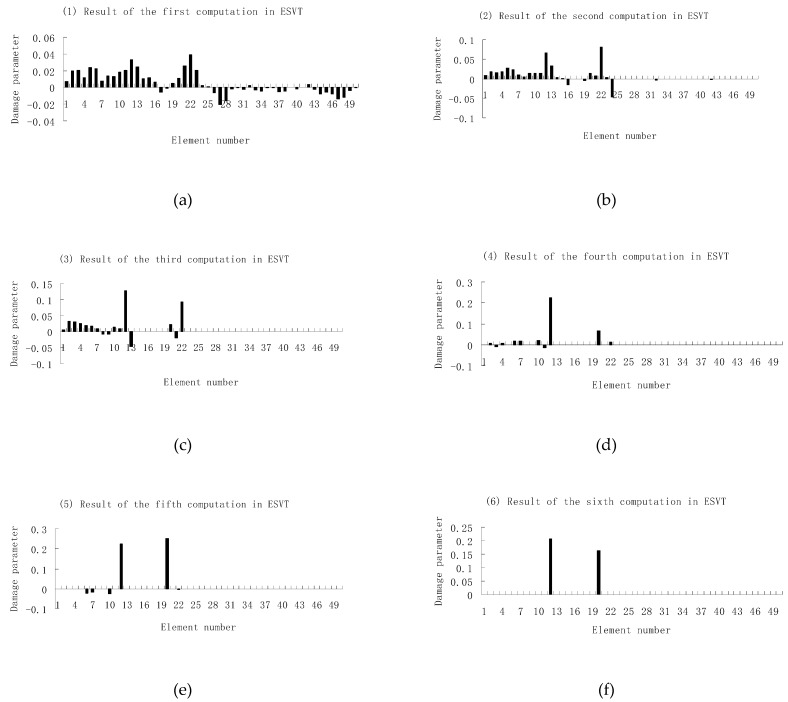
Damage evaluation results of the first to sixth computations in ESVT for the plate structure. (**a**) Result of the first computation in ESVT; (**b**) Result of the second computation in ESVT; (**c**) Result of the third computation in ESVT; (**d**) Result of the fourth computation in ESVT; (**e**) Result of the fifth computation in ESVT; (**f**) Result of the sixth computation in ESVT.

A plate structure as shown in Figure 18 was used as the second example to verify the proposed method. The main purpose of using this example was to verify the effectiveness of the proposed method for structures that require solid finite elements. The modulus of elasticity, mass density, and Poisson’s ratio of this steel material were 200 GPa, 7800 kg/m^3^, and 0.3, respectively. The plate was modeled using 50 identical solid elements as shown in Figure 18. In the following damage simulation, it was assumed that elements 12 and 20 had 20% and 15% stiffness reductions, respectively. Using the first eight frequencies, damage evaluation results obtained by the proposed ESVT method are shown in Figure 19. One can see from Figure 19 that the solution accuracy of the first to sixth computations was improving gradually and the sixth solution was the final result. The final result in Figure 19 clearly indicated that elements 12 and 20 were the true damaged elements. These results show that the proposed ESVT method can also be used successfully in structures that require solid finite elements.

## 4. Experimental Validation

**Figure 20:**
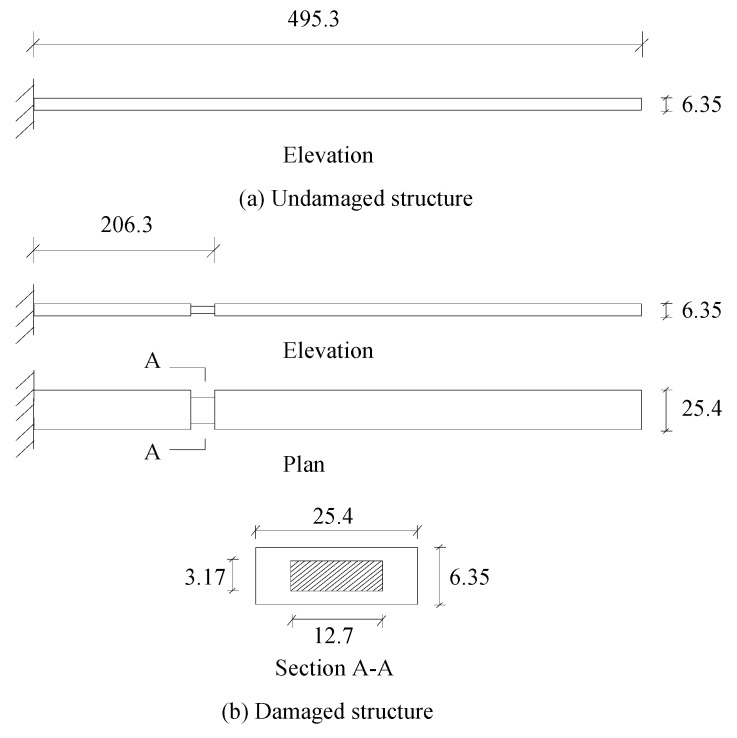
Configuration of the experimental beam [37]. (**a**) Undamaged structure; (**b**) Damage structure.

In this section, the experimental beam conducted by Yang et al. [37] was used as an example to verify the proposed method. As shown in Figure 20a, the length, width and height of the intact beam were 495.3 mm, 25.4 mm and 6.35 mm, respectively. The modulus of elasticity and mass density of this aluminium material were 71 GPa and 2210 kg/m^3^, respectively. The beam was modeled using 20 equal-length elements and the damage was induced in the ninth element by a saw cut as shown in Figure 20b. The analytical and experimental values of the first six natural frequencies for the undamaged and damaged structures are all shown in Table 2.

**Figure 21:**
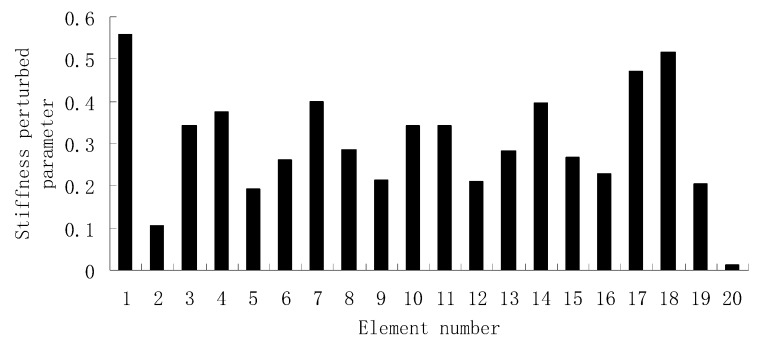
The stiffness perturbed parameters of the modified finite element model (FEM).

**Figure 22:**
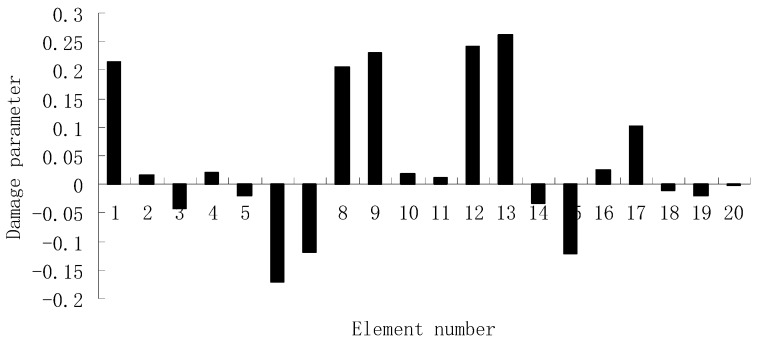
Damage evaluation result by the first computation of ESVT for the experimental beam.

**Figure 23:**
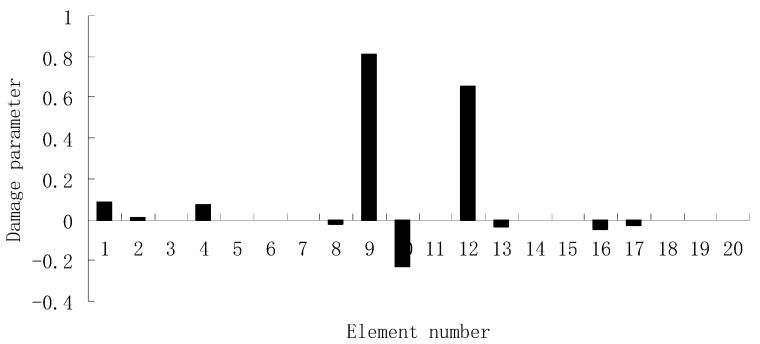
Damage evaluation result by the second computation of ESVT for the experimental beam.

**Figure 24:**
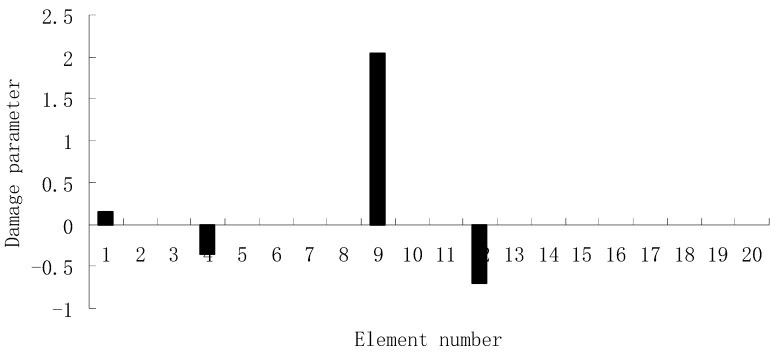
Damage evaluation result by the third computation of ESVT for the experimental beam.

**Figure 25:**
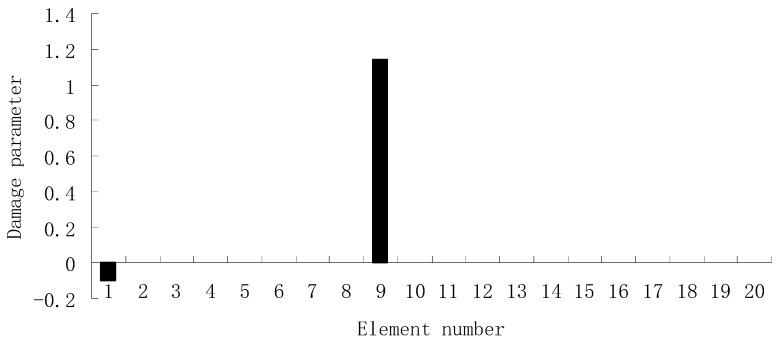
Damage evaluation result by the fourth computation of ESVT for the experimental beam.

**Figure 26:**
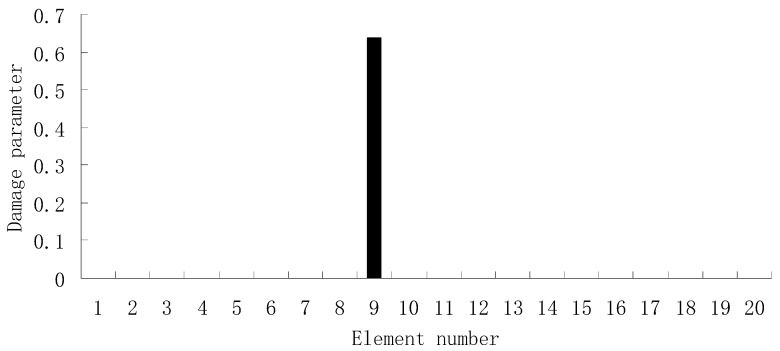
Damage evaluation result by the fifth computation of ESVT for the experimental beam.

**Figure 27:**
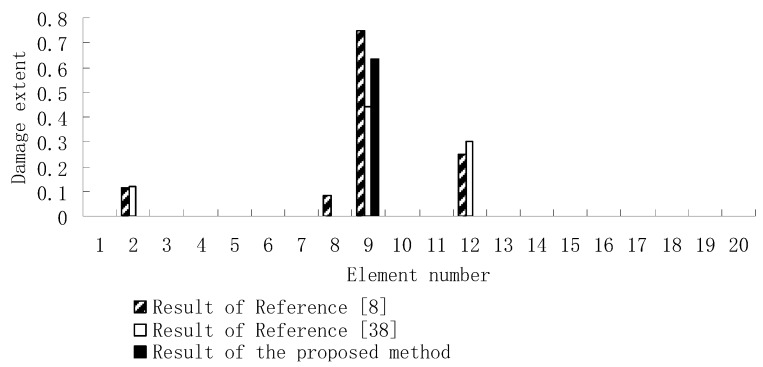
Comparison of damage evaluation results by the three methods.

From columns 2 and 3 in Table 2, one can see that the differences between the analytical values obtained by FEM and the experimental values obtained by dynamic testing of the undamaged beam were very large. This means that the original FEM constructed by the software was not accurate enough to represent the undamaged beam. Thus the FEM of the undamaged beam was firstly corrected according to the natural frequency changes between the analytical values and the undamaged experimental values. Only the modified FEM could be used in the subsequent evaluation of structural damage. Note that the natural frequency sensitivity technique introduced in Section 2.1 can be used not only in damage evaluation but also in model updating. It should also be noted that the stiffness perturbed parameters of the modified FEM were computed only by one calculation process of the ESVT method in the model updating. This is the difference between the model updating problem and the damage identification problem. From the variations between column 2 and column 3 in Table 2, Figure 21 presents the stiffness perturbed parameters of the modified FEM and Table 3 gives the analytical values of the first six natural frequencies obtained by the modified FEM. From Table 3, one can see that the analytical values of the modified FEM were much closer to the undamaged experimental values than those of the original FEM. After model updating, structural damage evaluation can be subsequently carried out based on the modified FEM by using the gradual ESVT method. Figure 22, Figure 23, Figure 24, Figure 25 and Figure 26 give the damage evaluation results of the first to fifth calculations in the ESVT. It was obvious that the accuracy of damage evaluation result in Figure 22, Figure 23, Figure 24, Figure 25 and Figure 26 was improving gradually and Figure 26 was the final evaluation result. The final evaluation result of Figure 26 was very good since the true damage was correctly detected in element 9. For comparisons, the damage detection results reported by Krishnanunni et al. [8] and Hao et al. [38] are presented in Figure 27, obtained by Cuckoo Search algorithm (CSA) and Genetic algorithm (GA), respectively. Meanwhile, the result of Figure 26 is also shown in Figure 27 for easy comparison. From Figure 27, one can see that the damage evaluation result obtained by the proposed ESVT method had the highest accuracy among the three methods. Moreover, the computational complexity of the ESVT method was significantly lower compared to the other methods because both CSA and GA needed many iterations for good convergence. For example, the computation process using CSA reported by Krishnanunni et al. [8] was iterated 65,000 times for good convergence. Note that the proposed ESVT method only needed five calculations and the complexity of each calculation decreased gradually.

## 5. Conclusions

For the damage evaluation problem, the damaged elements in the structure are often only a small minority because the actual damage usually occurs only in a few local areas. Using this particularity of damage evaluation, an ESVT method was proposed in this paper for structural damage detection using only a few lower order natural frequencies. Central to the ESVT method is the normalization of linear systems of equations and multiple computations based on feedback evaluation. In each computation of ESVT, many undamaged elements are removed according to the feedback evaluation to reduce the number of unknowns. This operation can significantly reduce the computational complexity and obtain more accurate damage evaluation results. The proposed method is very concise in theory and simple to implement. Two numerical examples and an experimental example were used to demonstrate the proposed method. From the numerical examples, it was found that the proposed method can successively obtain more accurate damage evaluation results compared with the traditional SVT method. From the experimental example, it was shown that the proposed method possesses more precise and fewer calculations compared with the existing optimization algorithms. It was shown that the proposed ESVT method may be a promising technique in non-destructive evaluation of structural damage. In practical applications, the proposed method can be applied to various types of structural damage such as reduction in elastic modulus and cracks, as long as these damages can cause observable frequency changes. Specific examples of crack detection by the proposed method will be further studied in the future.

## Figures and Tables

**Table 1:** The first six natural frequencies of the undamaged and damaged truss structures.

Natural Frequencies	Undamaged Structure	Damage Case 1	Damage Case 2
1	24.034	23.8588	23.9621
2	119.9908	119.3298	117.8529
3	195.9065	193.7641	194.9649
4	274.2121	273.7942	270.9942
5	436.9691	436.5461	436.3045
6	569.0272	568.812	565.0954

**Table 2:** The first six natural frequencies of the undamaged and damaged beams [37].

Natural Frequencies	Analytical Values (Hz)	Experimental Values (Undamaged)	Experimental Values (Damaged)
1	23.7	19.53	19.00
2	148.5	122.05	115.85
3	415.7	339.26	332.36
4	814.2	661.73	646.91
5	1345.3	1085.22	1037.46
6	2008.7	1594.59	1591.36

**Table 3:** Comparisons of natural frequencies obtained by the original FEM, the modified FEM and the experiment.

Natural Frequencies	Analytical Values of Original FEM (Hz)	Experimental Values (Undamaged Beam)	Analytical Values of Modified FEM (Hz)
1	23.7 (21.4%^*^)	19.53	19.0 (2.7%)
2	148.5 (21.7%)	122.05	119.8 (1.8%)
3	415.7 (22.5%)	339.26	333.7 (1.6%)
4	814.2 (23.0%)	661.73	651.2 (1.6%)
5	1345.3 (24.0%)	1085.22	1068.7 (1.5%)
6	2008.7 (26.0%)	1594.59	1582.6 (0.8%)

* The data in brackets denote the relative errors between analytical and experimental values.

## References

[1] Salawu O.S. (1997). Detection of structural damage through changes in frequency: a review. Eng. Struct..

[2] Yang Q.W., Liu J.K. (2006). A coupled method for structural damage identification. J. Sound Vib..

[3] Messina A., Williams J.E., Contursi T. (1996). Structural damage detection by a sensitivity and statistical-based method. J. Sound Vib..

[4] Yu L., Cheng L., Yam L.H., Yan Y.J. (2007). Application of eigenvalue perturbation theory for detecting small structural damage using dynamic responses. Compos. Struct..

[5] Yang Q.W., Liu J.K. (2009). Structural damage identification by adding given masses. Eng. Mech..

[6] Khiem N.T., Toan L.K. (2014). A novel method for crack detection in beam-like structures by measurements of natural frequencies. J. Sound Vib..

[7] Ding Z., Lu Z., Huang M., Liu J. (2017). Improved artificial bee colony algorithm for crack identification in beam using natural frequencies only. Inverse Prob. Sci. Eng..

[8] Krishnanunni C.G., Raj R.S., Nandan D., Midhun C.K., Sajith A.S., Ameen M. (2019). Sensitivity-based damage detection algorithm for structures using vibration data. J. Civ. Struct. Health Monit..

[9] Choi A.J., Han J.H. (2018). Frequency-based damage detection in cantilever beam using vision-based monitoring system with motion magnification technique. J. Intell. Mater. Syst. Struct..

[10] Pan J., Zhang Z., Wu J., Ramakrishnan K.R., Singh H.K. (2019). A novel method of vibration modes selection for improving accuracy of frequency-based damage detection. Composites Part B.

[11] Ercolani G.D., Felix D.H., Ortega N.F. (2018). Crack detection in prestressed concrete structures by measuring their natural frequencies. J. Civ. Struct. Health Monit..

[12] Bicanic N., Chen H.P. (1997). Damage identification in framed structures using natural frequencies. Int. J. Numer. Methods Eng..

[13] Xia Y., Hao H., Brownjohn J.M.W., Xia P.Q. (2002). Damage identification of structures with uncertain frequency and mode shape data. Earthquake Eng. Struct. Dyn..

[14] Udwadia F.E. (2005). Structural identification and damage detection from noisy modal data. J. Aerosp. Eng..

[15] Yang J., Li P., Yang Y., Xu D. (2018). An improved EMD method for modal identification and a combined static-dynamic method for damage detection. J. Sound Vib..

[16] Rakha M.A. (2004). On the Moore–Penrose generalized inverse matrix. Appl. Math. Comput..

[17] Farrar C.R., Doebling S.W., Nix D.A. (2001). Vibration—Based structural damage identification. Philos. Trans. R. Soc. Lond. Ser. A.

[18] Ruggiero E.J., Park G., Inman D.J. (2004). Multi-input multi-output vibration testing of an inflatable torus. Mech. Syst. Sig. Process..

[19] Sodano H.A., Park G., Inman D.J. (2004). An investigation into the performance of macro-fiber composites for sensing and structural vibration applications. Mech. Syst. Sig. Process..

[20] Foti D., Diaferio M., Giannoccaro N.I., Mongelli M. (2012). Ambient vibration testing, dynamic identification and model updating of a historic tower. NDT and E Int..

[21] Neumaier A. (1998). Solving ill-conditioned and singular linear systems: A tutorial on regularization. SIAM Rev..

[22] Basseville M., Mevel L., Goursat M. (2004). Statistical model-based damage detection and localization: subspace-based residuals and damage-to-noise sensitivity ratios. J. Sound Vib..

[23] Chen H.P. (2008). Application of regularization methods to damage detection in large scale plane frame structures using incomplete noisy modal data. Eng. Struct..

[24] Weber B., Paultre P., Proulx J. (2009). Consistent regularization of nonlinear model updating for damage identification. Mech. Syst. Sig. Process..

[25] Li X.Y., Law S.S. (2010). Adaptive Tikhonov regularization for damage detection based on nonlinear model updating. Mech. Syst. Sig. Process..

[26] Huang Q., Gardoni P., Hurlebaus S.A. (2012). probabilistic damage detection approach using vibration-based nondestructive testing. Struct. Saf..

[27] Zhao Z., Lin R., Meng Z., He G., You L., Zhou Y. (2018). A modified truncation singular value decomposition method for solving ill-posed problems. J. Algorithms Comput. Technol..

[28] Arcucci R., Mottet L., Pain C., Guo Y.K. (2019). Optimal reduced space for Variational Data Assimilation. J. Comput. Phys..

[29] Golub G.H., Van Loan C.F. (1980). An analysis of the total least squares problem. SIAM J. Numer. Anal..

[30] Markovsky I., Van Huffel S. (2007). Overview of total least-squares methods. Signal Process..

[31] Weng Y., Xiao W., Xie L. (2011). Total least squares method for robust source localization in sensor networks using TDOA measurements. Int. J. Distrib. Sens. Netw..

[32] Tong X., Jin Y., Li L. (2011). An improved weighted total least squares method with applications in linear fitting and coordinate transformation. J. Surv. Eng..

[33] Li S., Liu L., Liu Z., Wang G. (2019). A robust total Kalman filter algorithm with numerical evaluation. Surv. Rev..

[34] Hansen P.C. (1992). Analysis of discrete ill-posed problems by means of the L-curve. SIAM Rev..

[35] Hansen P.C., O’Leary D.P. (1993). The use of the L-curve in the regularization of discrete ill-posed problems. SIAM J. Sci. Comput..

[36] Reichel L., Sadok H. (2008). A new L-curve for ill-posed problems. J. Comput. Appl. Math..

[37] Yang J.C.S., Tsai T., PEVLIN V. (1985). Structural damage detection by the system identification technique. Shock Vibr. Bull..

[38] Hao H., Xia Y. (2002). Vibration-based damage detection of structures by genetic algorithm. J. Comput. Civ. Eng..

